# Pneumococcal Otogenic Meningitis Complicated With Pneumocephalus and Coma State

**DOI:** 10.7759/cureus.10917

**Published:** 2020-10-12

**Authors:** Despoina Liourdi, Pantelis Litsardopoulos, Dimitra Dimitropoulou, Adrianni Fatourou, Andreas A Argyriou

**Affiliations:** 1 Internal Medicine, General Hospital of Patras, Patras, GRC; 2 Neurology, General Hospital of Patras, Patras, GRC; 3 Radiology, General Hospital of Patras, Patras, GRC; 4 Neurology, General Hospital of Patras "Agios Andreas", Patras, GRC

**Keywords:** otogenic meningitis, pneumococcus, complication, pneumocephalus, coma

## Abstract

We herein describe the unusual case of a male patient with pneumococcal otogenic meningitis, which was complicated with non-traumatic pneumocephalus and coma, in the absence of head trauma or a neurosurgical procedure. The initiation of an aggressive, empirical scheme with wide-spectrum antibiotics was achieved to stop the progression of meningitis, pneumocephalus, and their underlying causes in this patient. We propose pathogenetic mechanisms to explain this life-threatening condition.

## Introduction

Bacterial meningitis represents a fatal condition that requires prompt diagnosis and early therapeutical intervention [1]. Pneumococcal otitis media may be complicated with meningitis in up to 30% of cases. Pneumococcal meningitis, representing 50% of all bacterial meningitis cases, could rarely be further complicated with pneumocephalus [1-2].

Pneumocephalus refers to the presence of gas or air in the cranial cavity as a result of trauma, neurosurgical procedures, tumors, necrotic lesions after radiotherapy, meningitis, and/or encephalitis caused by gas-producing organisms [3]. Complications of middle ear infections with pneumocephalus are unusually encountered. After obtaining informed consent for the release of personal information, we herein report on a comatose patient due to pneumococcal otogenic meningitis and non-traumatic pneumocephalus.

## Case presentation

A 62-year-old male was transferred early morning to our emergency wards in an acute severe comatose state with a Glasgow Coma Scale of 5/15 and was urgently intubated. His spouse reported that he had flu-like symptoms and low-grade fever (up to 37.2°C) for approximately a week while he also complained of a dull headache the previous day, while being fully alert, and received 500 mg bi.d of paracetamol. The patient was also receiving for the previous 10 days etoricoxib (90 mg/day) due to knee joint pain. His past medical history was otherwise unremarkable. There was no history of recent trauma or neurosurgical procedure.

During the physical examination, the patient was hypothermic (35.7°C) with normal blood pressure and heart rate. Oxygen saturation was 82% with rebreather (6 lt/min). Chest auscultation revealed coarse crepitations in both lung bases. Cardiac and abdominal examinations were normal. Neurological examination showed that both eye pupils were unresponsive to light. There was no evidence of cervical stiffness or other signs of meningeal irritation. Plantar reflexes were flexors.

Laboratory testing revealed an elevated white blood cell count (WBC) of 18.200/μL with neutrophilia (93.7%) predominance. C-reactive protein (CRP) was highly elevated (14.51 mg/dl; range 0-0.5 mg/dl). General biochemistry was normal. Urine and blood cultures were obtained and the patient had an urgent brain and chest computed tomography (CT) scan.

**Figure 1:**
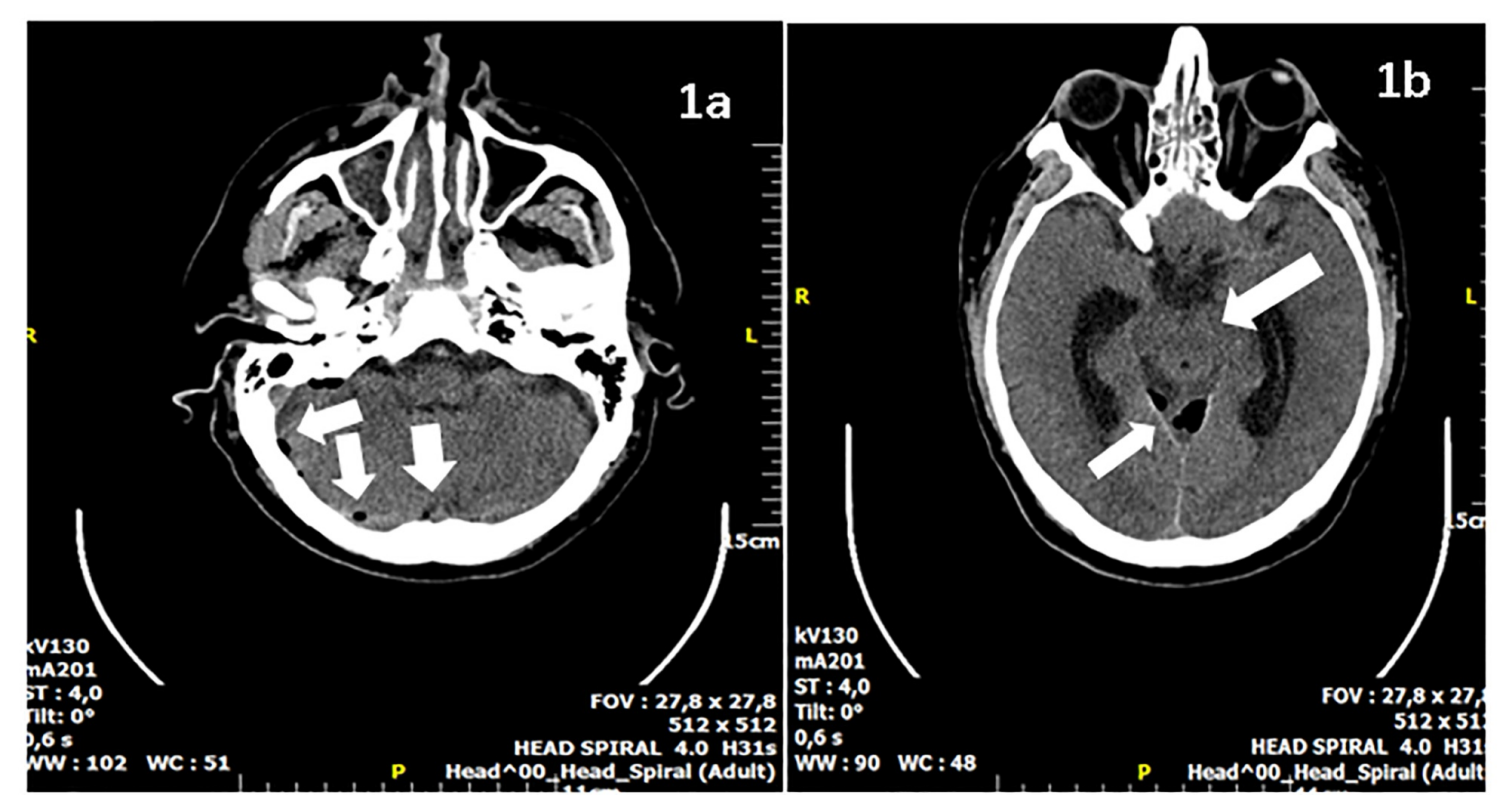
Axial CT brain 1A) Axial brain CT image showing pneumocephalus in the posterior cranial fossa; 1B) Axial brain CT image showing pneumocephalus at the level of the cerebellum in close relation to the right temporal and occipital bones. Diffuse edema of the brain stem and temporal lobes. CT: computed tomography

The brain CT scan revealed the presence of multiple intra-axial air bubbles scattered through several sites of the brain, the cerebellum, and the region of subarachnoid (quadrigeminal and interpeduncular) cisterns. A dilatation of both temporal horns of lateral ventricles, as well as of the third ventricle, was also noted. Loss of gray-white matter differentiation of the cerebellum and temporal lobes as also a subtly reduced density in the cerebrum, consistent with diffuse edema, were also present. There was no evidence of traumatic lesion, fracture, or congenital defect (Figures 1A-1B).

The chest CT scan documented ground-glass findings in both lungs while a consolidated upper right lobe was also observed. These are findings in keeping with aspiration pneumonia, which most probably occurred at the constellation of the acute coma.

Lumbar puncture was omitted at this time point due to the cerebral edema, and an empirical antibiotic scheme was initiated, comprising intravenous vancomycin (1 gr b.id), ceftriaxone (2 gr b.id), ampicillin (2 gr q.4 hr), metronidazole (500 mg t.id), and acyclovir (750 mg t.id). Dexamethasone (8 mg q.id) was also administered, and the patient was admitted to the intensive care unit (ICU). High-flow oxygen therapy was also commenced.

**Figure 2:**
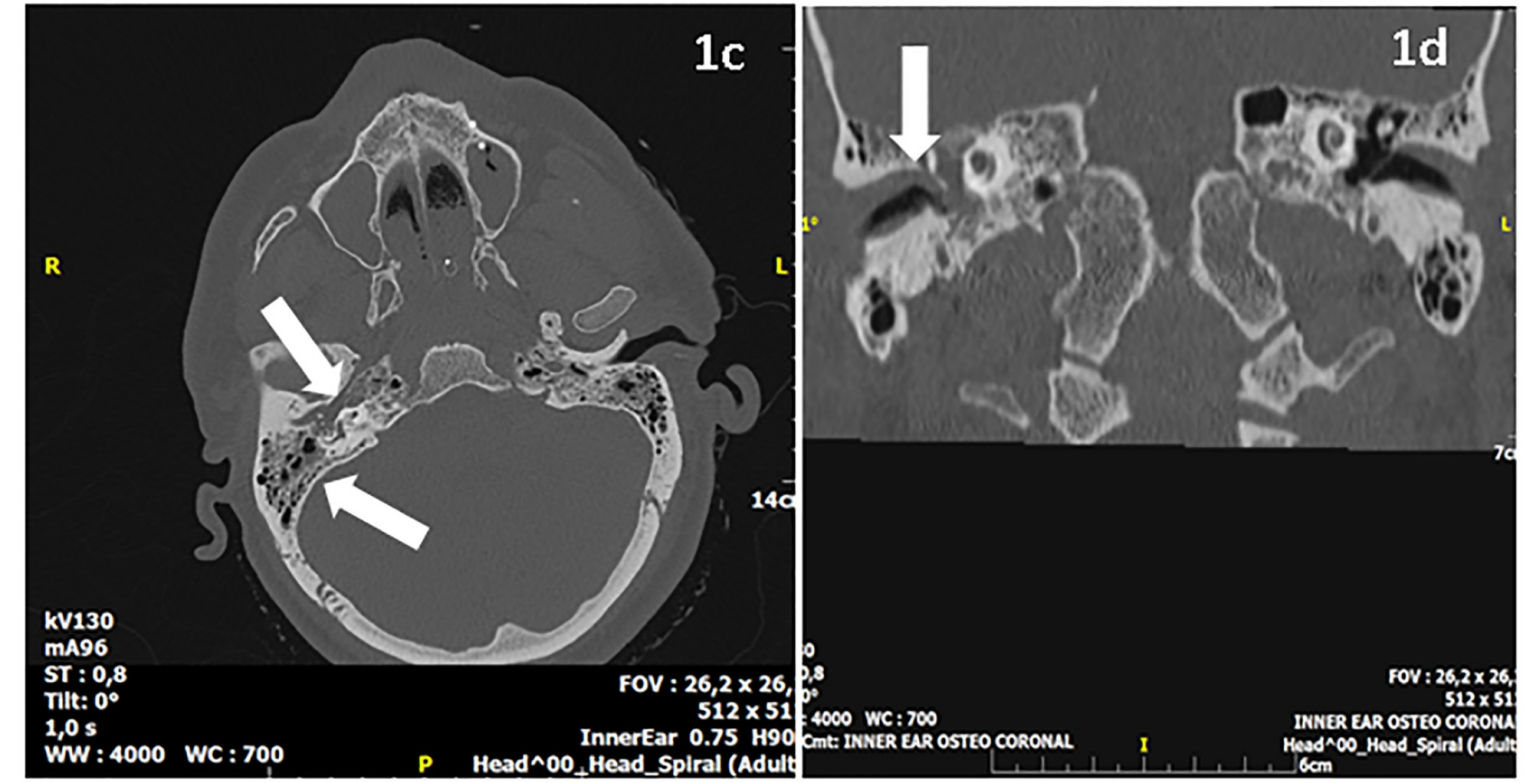
Axial brain and inner ear osteo-coronal CT images Axial brain and inner ear osteo-coronal CT images showing otitis media and mastoiditis on the right side. Presence of soft tissue density in the right middle ear cavity and the mastoid with hypo-pneumatization. CT: computed tomography

The WBC and CRP counts were increased to 23.200/μL and 30 mg/dl, respectively, on the next day, and ENT consultation recommended skull-base CT scan imaging, which documented silent right-sided otitis media and right-sided mastoiditis (Figure 2). 

A repeated brain CT scan three days later showed that both the pneumocephalus foci and edema had subsided, allowing for a spinal tap to be performed. Cerebrospinal fluid (CSF) analysis revealed evidence of highly elevated cell count (15.000/µL) with 85% polymorphonucleocytes while protein was increased (115 mg/dL; normal range 15-45). Gram staining was positive for gram-positive cocci. CSF and blood cultures yielded growth of Streptococcus pneumoniae, confirming as such the diagnosis of pneumococcal meningitis, and the antibiotics scheme was switched based on the findings of organism susceptibility. The patient was discharged after one month of hospitalization and, presently, he is fully recovered without any neurological sequelae.

## Discussion

The use of wide-spectrum antibiotic schemes has significantly lowered the incidence of the intracranial complications of otitis media, including bacterial meningitis, brain abscess, and lateral sinus thrombosis [4]. The diagnosis of pneumocephalus associated with pneumococcal meningitis is an unusual condition with only very few cases reported in the literature [2,5-9]. Even more rarely reported are cases with a relevant life-threatening phenotype.

Our case manifested severe sepsis and coma due to meningitis and pneumocephalus of silent streptococcal otogenic origin. Notably, there was no preceding neurological or ear symptomatology according to the history obtained by his family members, although, tellingly, the non-steroidal anti-inflammatory drugs (NSAIDs) intake for 10 days could have masked the typical findings of bacterial meningitis, including severe headache, pyrexia, otalgia, and meningeal signs [10].

Despite the atypical or masked presentation, neuroimaging with a brain CT scan strongly supported the diagnosis of infectious otogenic meningitis and pneumocephalus, which was confirmed by CSF and blood cultures. The use of a CT scan is preferred over the use of magnetic resonance imaging (MRI) in the diagnosis of pneumocephalus, as it can detect even 0.55 ml of intracranial air, whereas the presence of air in MRI may be perceived wrongly for flow voids or blood products [11].

Delays in the prompt diagnosis of this potentially severe condition and a thorough initiation of antibiotics have been linked to increased mortality [2]. In line with this, our patient was admitted in a severe coma state and evidence of brain edema on imaging. Indeed, pneumocephalus can occasionally behave like a space-occupying lesion with triggering of intracranial hypertension leading to brain edema and life-threatening conditions [10]. On the basis of the latter evidence, a potent wide-spectrum empirical antibiotic scheme was promptly administered, which was life-saving, as it soon achieved the stopping of the progression of meningitis, pneumocephalus, and their underlying causes in our patient, including silent medial otitis and mastoiditis at the right side. To our knowledge, the literature contains only one similar case, which had a much more benign clinical phenotype and excellent outcome than ours [12].

Attempting to explain the pathophysiological background of meningitis genesis in our case, one could blame the dissemination of the gas-forming bacteria, which could exist in the otogenic foci [13]. However, considering that streptococcus pneumoniae is not a typical gas-producing bacteria, we suggest that its direct invasion from the middle ear, with the destruction of the leptomeninges and skull, might hold the response [14]. On the other hand, pneumocephalus could have been generated from streptococcal hematogenous dissemination in the posterior cranial fossa through small vessels connecting the vascular networks of the temporal bone, dura, and venous sinuses [5]. The high-flow oxygen supplementation we applied increased the nitrogen concentration gradient to facilitate absorption between the air collection and surrounding brain tissue.

## Conclusions

Summarizing existing data, our case presented meningitis and pneumocephalus of silent streptococcal otogenic origin, which was further complicated with a severe coma. As such, physicians, particularly those working in the emergency setting, should be aware of potential serious otogenic neurological complications, even in the absence of typical symptoms and signs. We suggest that our report is clinically significant because it highlights the need for applying thorough history-taking, examination, and imaging in order to obtain the diagnosis early and to commence appropriate aggressive treatment with antibiotics. In addition, the presence of pneumocephalus without previous trauma in meningoencephalitis patients is highly suggestive of a tentative diagnosis of ear infection, thoroughly needing an urgent ENT examination.
